# 7-Bromo-1-(4-fluoro­phenyl­sulfon­yl)-2-methyl­naphtho­[2,1-*b*]furan

**DOI:** 10.1107/S160053681005436X

**Published:** 2011-01-08

**Authors:** Hong Dae Choi, Pil Ja Seo, Byeng Wha Son, Uk Lee

**Affiliations:** a Department of Chemistry, Dongeui University, San 24 Kaya-dong Busanjin-gu, Busan 614-714, Republic of Korea; b Department of Chemistry, Pukyong National University, 599-1 Daeyeon 3-dong, Nam-gu, Busan 608-737, Republic of Korea

## Abstract

In the title compound, C_19_H_12_BrFO_3_S, the 4-fluoro­phenyl ring makes a dihedral angle of 80.32 (5)° with the mean plane of the naphtho­furan fragment. In the crystal, mol­ecules are linked by weak inter­molecular C—H⋯O and C—H⋯π inter­actions. The crystal structure also exhibits aromatic π–π inter­actions between the central benzene rings of neighbouring mol­ecules [centroid–centroid distance = 3.564 (3) Å].

## Related literature

For the pharmacological activity of naphtho­furan compounds, see: Einhorn *et al.* (1984 ▶); Hranjec *et al.* (2003 ▶); Mahadevan & Vaidya (2003 ▶). For our previous structural studies of related 1-aryl­sulfonyl-7-bromo-2-methyl­naphtho­[2,1-*b*]furan derivatives, see: Choi *et al.* (2008*a* ▶,*b* ▶).
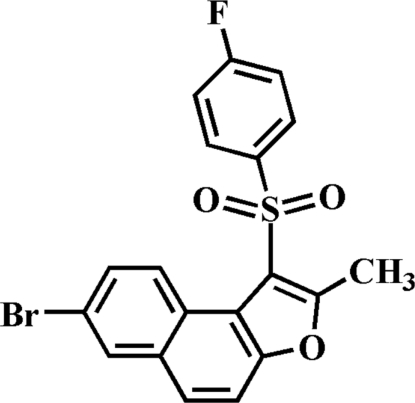


## Experimental

### 

#### Crystal data

C_19_H_12_BrFO_3_S
*M* *_r_* = 419.26
Monoclinic, 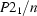

*a* = 12.0415 (2) Å
*b* = 8.1579 (1) Å
*c* = 17.4578 (3) Å
β = 105.325 (1)°
*V* = 1653.96 (5) Å^3^
*Z* = 4
Mo *K*α radiation
μ = 2.64 mm^−1^
*T* = 173 K
0.25 × 0.21 × 0.12 mm

#### Data collection

Bruker SMART APEXII CCD diffractometer
Absorption correction: multi-scan (*SADABS*; Bruker, 2009 ▶) *T*_min_ = 0.553, *T*_max_ = 0.744
14803 measured reflections
3803 independent reflections
3026 reflections with *I* > 2σ(*I*)
*R*_int_ = 0.032

#### Refinement

*R*[*F* ^2^ > 2σ(*F* ^2^)] = 0.031
*wR*(*F* ^2^) = 0.078
*S* = 1.02
3803 reflections
227 parameters
H-atom parameters constrained
Δρ_max_ = 0.42 e Å^−3^
Δρ_min_ = −0.41 e Å^−3^

### 

Data collection: *APEX2* (Bruker, 2009 ▶); cell refinement: *SAINT* (Bruker, 2009 ▶); data reduction: *SAINT*; program(s) used to solve structure: *SHELXS97* (Sheldrick, 2008 ▶); program(s) used to refine structure: *SHELXL97* (Sheldrick, 2008 ▶); molecular graphics: *ORTEP-3* (Farrugia, 1997 ▶) and *DIAMOND* (Brandenburg, 1998 ▶); software used to prepare material for publication: *SHELXL97*.

## Supplementary Material

Crystal structure: contains datablocks global, I. DOI: 10.1107/S160053681005436X/rk2255sup1.cif

Structure factors: contains datablocks I. DOI: 10.1107/S160053681005436X/rk2255Isup2.hkl

Additional supplementary materials:  crystallographic information; 3D view; checkCIF report

## Figures and Tables

**Table 1:** Hydrogen-bond geometry (Å, °) *Cg*1 is the centroid of the C14–C19 4-fluoro­phenyl ring.

*D*—H⋯*A*	*D*—H	H⋯*A*	*D*⋯*A*	*D*—H⋯*A*
C5—H5⋯O2^i^	0.95	2.57	3.312 (3)	135
C18—H18⋯O2^ii^	0.95	2.36	3.266 (3)	160
C19—H19⋯O3^i^	0.95	2.57	3.113 (2)	117
C10—H10⋯*Cg*1^iii^	0.95	2.69	3.609 (2)	152

Symmetry codes: (i) 
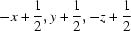
; (ii) 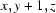; (iii) 
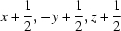
.

**Fig. 1.**
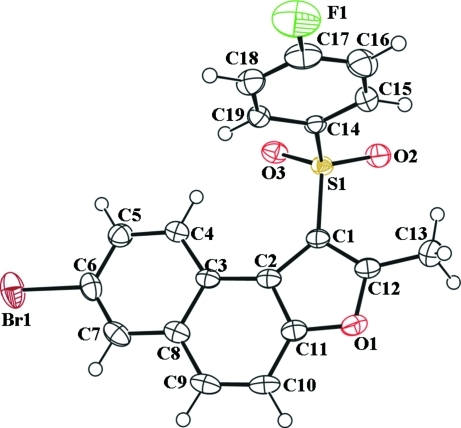
The molecular structure of the title compound with the atom numbering scheme. Displacement ellipsoids are drawn at the 50% probability level. H atoms are presented as a small spheres of arbitrary radius.

**Fig. 2.**
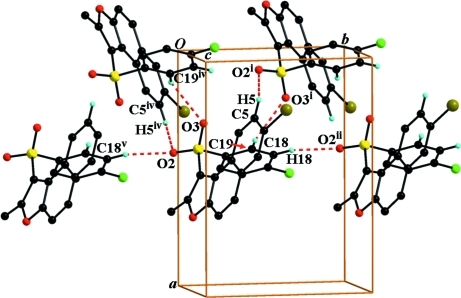
A view of C–H···O interactions (dotted lines) in the crystal structure of the title compound. Symmetry codes: (i) -x+1/2, y+1/2, -z+1/2; (ii) x, y+1, z; (iv) -x+1/2, y-1/2, -z+1/2; (v) x, y-1, z.

**Fig. 3.**
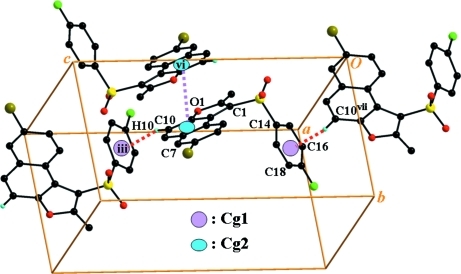
A view of C–H···π and π–π interactions (dotted lines) in the crystal structure of the title compound. Symmetry codes: (iii) x+1/2, -y+1/2, z+1/2; (vi) -x+1, -y, -z + 1; (vii) x-1/2, -y+1/2, z-1/2.

## supplementary crystallographic information

### Comment

Many compounds involving a naphthofuran ring have attracted much attention in view of their diverse pharmacological properties such as antibacterial, antitumor and anthelmintic activities (Einhorn *et al.*, 1984, Hranjec *et al.*, 2003, Mahadevan & Vaidya, 2003). As a part of our ongoing studies of the substituent effect on the solid state structures of 1-arylsulfonyl-7-bromo-2-methylnaphtho[2,1-*b*]furan analogues (Choi *et al.*, 2008**a*,b*), we report herein on the crystal structure of the title compound.

In the title molecule (Fig. 1), the naphthofuran moiety is essentially planar, with a mean deviation of 0.011 (2)Å from the least-squares plane defined by the thirteen constituent atoms. The dihedral angle formed by the mean plane of the naphthofuran system and the 4-fluorophenyl ring is 80.32 (5)°. The crystal packing (Fig. 2) is stabilized by weak intermolecular C–H···O hydrogen bonds; the first one between a benzene H atom and the oxygen of the O═S═O unit (Table 1; C5–H5···O2^i^), and the second one between the 4-fluorophenyl H atom and the oxygen of the O═S═O unit (Table 1; C18–H18···O2^ii^), and the third one between the 4-fluorophenyl H and the oxygen of the O═S═O unit (Table 1; C19–H19···O3^i^). The molecular packing (Fig. 3) is further stabilized by an intermolecular C–H···π interaction between a benzene H atom and the 4-fluorophenyl ring (Table 1; C10–H10···Cg1^iii^, Cg1 is the centroid of the C14-C19 4-fluorophenyl ring). In addition, the crystal packing (Fig. 3) exhibits an aromatic π–π interaction between the central benzene rings of neighbouring molecules. The Cg2···Cg2^vi^ distance is 3.564 (3)Å (Cg2 is the centroid of the C2/C3/C8-C11 benzene ring).

### Experimental

3-Chloroperoxybenzoic acid (77%) (404 mg, 1.8 mmol) was added in small portions to a stirred solution of 7-bromo-1-(4-fluorophenylsulfanyl)-2-methylnaphtho[2,1-b]furan (348 mg, 0.9 mmol) in dichloromethane (40 mL) at 273 K. After being stirred at room temperature for 10 h, the mixture was washed with saturated sodium bicarbonate solution and the organic layer was separated, dried over magnesium sulfate, filtered and concentrated at reduced pressure. The residue was purified by column chromatography (hexane-ethyl acetate, 4:1 v/v) to afford the title compound as a colourless solid [yield 73%, m.p. 485-486 K; R_f_ = 0.52 (hexane-ethyl acetate, 4:1 v/v)]. Single crystals suitable for X-ray diffraction were prepared by slow evaporation of a solution of the title compound in acetone at room temperature.

### Refinement

All H atoms were positioned geometrically and refined using a riding model, with C–H = 0.95Å for aryl and 0.98Å for methyl H atoms. *U*_iso_(H) = 1.2*U*_eq_(C) for aryl and 1.5*U*_eq_(C) for methyl H atoms.

### Crystal data

**Table:**

C_19_H_12_BrFO_3_S	*F*(000) = 840
*M**_r_* = 419.26	*D*_x_ = 1.684 Mg m^−^^3^
Monoclinic, *P*2_1_/*n*	Melting point = 485–486 K
Hall symbol: -P 2yn	Mo *K*α radiation, λ = 0.71073 Å
*a* = 12.0415 (2) Å	Cell parameters from 4448 reflections
*b* = 8.1579 (1) Å	θ = 2.4–27.4°
*c* = 17.4578 (3) Å	µ = 2.64 mm^−^^1^
β = 105.325 (1)°	*T* = 173 K
*V* = 1653.96 (5) Å^3^	Block, colourless
*Z* = 4	0.25 × 0.21 × 0.12 mm

### Data collection

**Table:**

Bruker SMART APEXII CCD diffractometer	3803 independent reflections
Radiation source: rotating anode	3026 reflections with *I* > 2σ(*I*)
graphite multilayer	*R*_int_ = 0.032
Detector resolution: 10.0 pixels mm^-1^	θ_max_ = 27.6°, θ_min_ = 1.9°
φ and ω scans	*h* = −15→15
Absorption correction: multi-scan (*SADABS*; Bruker, 2009)	*k* = −10→10
*T*_min_ = 0.553, *T*_max_ = 0.744	*l* = −22→22
14803 measured reflections	

### Refinement

**Table:**

Refinement on *F*^2^	Primary atom site location: structure-invariant direct methods
Least-squares matrix: full	Secondary atom site location: difference Fourier map
*R*[*F*^2^ > 2σ(*F*^2^)] = 0.031	Hydrogen site location: inferred from neighbouring sites
*wR*(*F*^2^) = 0.078	H-atom parameters constrained
*S* = 1.02	*w* = 1/[σ^2^(*F*_o_^2^) + (0.0385*P*)^2^ + 0.6504*P*] where *P* = (*F*_o_^2^ + 2*F*_c_^2^)/3
3803 reflections	(Δ/σ)_max_ = 0.001
227 parameters	Δρ_max_ = 0.42 e Å^−^^3^
0 restraints	Δρ_min_ = −0.41 e Å^−^^3^

### Special details

**Table:**

Geometry. All s.u.'s (except the s.u. in the dihedral angle between two l.s. planes) are estimated using the full covariance matrix. The cell s.u.'s are taken into account individually in the estimation of s.u.'s in distances, angles and torsion angles; correlations between s.u.'s in cell parameters are only used when they are defined by crystal symmetry. An approximate (isotropic) treatment of cell s.u.'s is used for estimating s.u.'s involving l.s. planes.
Refinement. Refinement of *F*^2^ against ALL reflections. The weighted *R*-factor w*R* and goodness of fit *S* are based on *F*^2^, conventional *R*-factors *R* are based on *F*, with *F* set to zero for negative *F*^2^. The threshold expression of *F*^2^ > 2σ(*F*^2^) is used only for calculating *R*-factors(gt) etc. and is not relevant to the choice of reflections for refinement. *R*-factors based on *F*^2^ are statistically about twice as large as those based on *F*, and *R*-factors based on ALL data will be even larger.

### Fractional atomic coordinates and isotropic or equivalent isotropic displacement parameters (Å^2^)

**Table:**

	*x*	*y*	*z*	*U*_iso_*/*U*_eq_	
Br1	0.23409 (2)	0.55058 (3)	0.595181 (15)	0.04881 (10)	
S1	0.40951 (4)	0.07887 (6)	0.25320 (3)	0.02349 (12)	
F1	0.52925 (15)	0.6513 (2)	0.09244 (10)	0.0670 (5)	
O1	0.68915 (11)	0.0164 (2)	0.42360 (8)	0.0308 (3)	
O2	0.42383 (14)	−0.06341 (18)	0.20874 (9)	0.0363 (4)	
O3	0.29924 (11)	0.1077 (2)	0.26702 (8)	0.0316 (3)	
C1	0.51536 (16)	0.0748 (2)	0.34372 (11)	0.0240 (4)	
C2	0.52174 (16)	0.1490 (3)	0.42090 (10)	0.0234 (4)	
C3	0.44999 (16)	0.2428 (2)	0.45819 (10)	0.0236 (4)	
C4	0.33609 (16)	0.2927 (3)	0.42204 (11)	0.0285 (4)	
H4	0.3021	0.2637	0.3682	0.034*	
C5	0.27304 (18)	0.3815 (3)	0.46198 (12)	0.0327 (5)	
H5	0.1963	0.4132	0.4363	0.039*	
C6	0.32294 (19)	0.4253 (3)	0.54121 (13)	0.0322 (5)	
C7	0.43192 (19)	0.3815 (3)	0.57901 (12)	0.0329 (5)	
H7	0.4638	0.4137	0.6326	0.040*	
C8	0.49895 (17)	0.2882 (3)	0.53949 (11)	0.0277 (4)	
C9	0.61271 (18)	0.2411 (3)	0.58063 (11)	0.0331 (5)	
H9	0.6429	0.2747	0.6342	0.040*	
C10	0.67917 (17)	0.1498 (3)	0.54546 (12)	0.0323 (5)	
H10	0.7547	0.1165	0.5731	0.039*	
C11	0.63028 (16)	0.1071 (3)	0.46594 (11)	0.0269 (4)	
C12	0.61837 (17)	−0.0014 (3)	0.34912 (12)	0.0280 (4)	
C13	0.6701 (2)	−0.0921 (3)	0.29391 (14)	0.0407 (6)	
H13A	0.7321	−0.0267	0.2826	0.061*	
H13B	0.6110	−0.1136	0.2443	0.061*	
H13C	0.7014	−0.1964	0.3182	0.061*	
C14	0.44463 (15)	0.2512 (3)	0.20340 (10)	0.0219 (4)	
C15	0.49975 (17)	0.2294 (3)	0.14369 (11)	0.0327 (5)	
H15	0.5180	0.1225	0.1292	0.039*	
C16	0.5279 (2)	0.3660 (4)	0.10549 (13)	0.0423 (6)	
H16	0.5651	0.3549	0.0641	0.051*	
C17	0.5005 (2)	0.5172 (3)	0.12903 (14)	0.0400 (6)	
C18	0.44494 (19)	0.5411 (3)	0.18684 (13)	0.0344 (5)	
H18	0.4264	0.6484	0.2007	0.041*	
C19	0.41645 (17)	0.4054 (3)	0.22448 (11)	0.0268 (4)	
H19	0.3775	0.4180	0.2649	0.032*	

### Atomic displacement parameters (Å^2^)

**Table:**

	*U*^11^	*U*^22^	*U*^33^	*U*^12^	*U*^13^	*U*^23^
Br1	0.06439 (18)	0.04182 (17)	0.05384 (16)	−0.00541 (13)	0.03959 (13)	−0.00784 (12)
S1	0.0259 (2)	0.0209 (3)	0.0210 (2)	−0.00233 (19)	0.00152 (17)	−0.00073 (19)
F1	0.0806 (11)	0.0562 (11)	0.0654 (10)	−0.0212 (9)	0.0216 (9)	0.0257 (9)
O1	0.0236 (7)	0.0369 (9)	0.0295 (7)	0.0027 (6)	0.0025 (6)	0.0064 (7)
O2	0.0475 (9)	0.0228 (8)	0.0324 (7)	0.0016 (7)	0.0001 (7)	−0.0057 (7)
O3	0.0245 (7)	0.0399 (9)	0.0282 (7)	−0.0059 (7)	0.0031 (5)	0.0003 (7)
C1	0.0260 (9)	0.0230 (11)	0.0215 (8)	−0.0011 (8)	0.0035 (7)	0.0048 (8)
C2	0.0254 (9)	0.0204 (10)	0.0220 (8)	−0.0053 (8)	0.0019 (7)	0.0044 (8)
C3	0.0282 (9)	0.0200 (10)	0.0208 (8)	−0.0053 (8)	0.0034 (7)	0.0045 (8)
C4	0.0303 (10)	0.0307 (12)	0.0235 (9)	−0.0016 (9)	0.0054 (8)	0.0011 (9)
C5	0.0320 (10)	0.0350 (13)	0.0316 (10)	0.0018 (10)	0.0095 (8)	0.0037 (10)
C6	0.0453 (12)	0.0250 (12)	0.0328 (10)	−0.0040 (10)	0.0217 (9)	−0.0015 (9)
C7	0.0460 (12)	0.0310 (12)	0.0233 (9)	−0.0120 (10)	0.0116 (9)	−0.0008 (9)
C8	0.0342 (10)	0.0240 (11)	0.0235 (9)	−0.0085 (9)	0.0049 (8)	0.0018 (9)
C9	0.0374 (11)	0.0355 (13)	0.0210 (9)	−0.0114 (10)	−0.0017 (8)	0.0019 (9)
C10	0.0262 (10)	0.0376 (13)	0.0263 (9)	−0.0054 (9)	−0.0048 (8)	0.0076 (10)
C11	0.0258 (9)	0.0260 (11)	0.0269 (9)	−0.0033 (8)	0.0037 (8)	0.0058 (9)
C12	0.0286 (10)	0.0283 (11)	0.0252 (9)	−0.0005 (9)	0.0036 (8)	0.0062 (9)
C13	0.0378 (12)	0.0487 (16)	0.0375 (12)	0.0106 (11)	0.0131 (9)	0.0027 (11)
C14	0.0200 (8)	0.0243 (11)	0.0187 (8)	0.0015 (8)	0.0004 (6)	0.0014 (8)
C15	0.0360 (11)	0.0347 (13)	0.0292 (10)	0.0052 (10)	0.0116 (9)	−0.0006 (10)
C16	0.0404 (12)	0.0553 (18)	0.0359 (11)	0.0010 (12)	0.0183 (10)	0.0100 (12)
C17	0.0396 (12)	0.0381 (14)	0.0387 (12)	−0.0108 (11)	0.0040 (10)	0.0151 (11)
C18	0.0394 (12)	0.0222 (12)	0.0366 (11)	−0.0024 (10)	0.0012 (9)	0.0004 (10)
C19	0.0283 (10)	0.0259 (11)	0.0242 (9)	0.0014 (9)	0.0036 (7)	−0.0016 (9)

### Geometric parameters (Å, °)

**Table:**

Br1—C6	1.900 (2)	C7—H7	0.9500
S1—O3	1.4303 (14)	C8—C9	1.421 (3)
S1—O2	1.4319 (16)	C9—C10	1.354 (3)
S1—C1	1.7487 (18)	C9—H9	0.9500
S1—C14	1.762 (2)	C10—C11	1.401 (3)
F1—C17	1.357 (3)	C10—H10	0.9500
O1—C12	1.361 (2)	C12—C13	1.477 (3)
O1—C11	1.369 (3)	C13—H13A	0.9800
C1—C12	1.368 (3)	C13—H13B	0.9800
C1—C2	1.461 (3)	C13—H13C	0.9800
C2—C11	1.378 (3)	C14—C19	1.378 (3)
C2—C3	1.433 (3)	C14—C15	1.387 (3)
C3—C4	1.410 (3)	C15—C16	1.386 (3)
C3—C8	1.434 (3)	C15—H15	0.9500
C4—C5	1.367 (3)	C16—C17	1.367 (4)
C4—H4	0.9500	C16—H16	0.9500
C5—C6	1.401 (3)	C17—C18	1.364 (3)
C5—H5	0.9500	C18—C19	1.376 (3)
C6—C7	1.352 (3)	C18—H18	0.9500
C7—C8	1.415 (3)	C19—H19	0.9500
			
O3—S1—O2	118.34 (10)	C9—C10—C11	116.25 (18)
O3—S1—C1	109.76 (9)	C9—C10—H10	121.9
O2—S1—C1	108.13 (9)	C11—C10—H10	121.9
O3—S1—C14	107.86 (9)	O1—C11—C2	112.00 (17)
O2—S1—C14	107.47 (9)	O1—C11—C10	121.84 (18)
C1—S1—C14	104.37 (9)	C2—C11—C10	126.2 (2)
C12—O1—C11	106.89 (15)	O1—C12—C1	110.17 (18)
C12—C1—C2	107.40 (16)	O1—C12—C13	114.29 (17)
C12—C1—S1	120.12 (15)	C1—C12—C13	135.52 (19)
C2—C1—S1	132.37 (15)	C12—C13—H13A	109.5
C11—C2—C3	117.91 (17)	C12—C13—H13B	109.5
C11—C2—C1	103.54 (17)	H13A—C13—H13B	109.5
C3—C2—C1	138.54 (17)	C12—C13—H13C	109.5
C4—C3—C2	125.66 (17)	H13A—C13—H13C	109.5
C4—C3—C8	117.73 (18)	H13B—C13—H13C	109.5
C2—C3—C8	116.61 (17)	C19—C14—C15	121.19 (19)
C5—C4—C3	122.05 (18)	C19—C14—S1	119.23 (14)
C5—C4—H4	119.0	C15—C14—S1	119.57 (17)
C3—C4—H4	119.0	C16—C15—C14	119.0 (2)
C4—C5—C6	119.2 (2)	C16—C15—H15	120.5
C4—C5—H5	120.4	C14—C15—H15	120.5
C6—C5—H5	120.4	C17—C16—C15	118.1 (2)
C7—C6—C5	121.5 (2)	C17—C16—H16	120.9
C7—C6—Br1	120.08 (16)	C15—C16—H16	120.9
C5—C6—Br1	118.42 (17)	F1—C17—C18	117.9 (2)
C6—C7—C8	120.63 (19)	F1—C17—C16	118.3 (2)
C6—C7—H7	119.7	C18—C17—C16	123.8 (2)
C8—C7—H7	119.7	C17—C18—C19	118.1 (2)
C7—C8—C9	119.80 (18)	C17—C18—H18	121.0
C7—C8—C3	118.90 (18)	C19—C18—H18	121.0
C9—C8—C3	121.30 (19)	C18—C19—C14	119.79 (19)
C10—C9—C8	121.73 (18)	C18—C19—H19	120.1
C10—C9—H9	119.1	C14—C19—H19	120.1
C8—C9—H9	119.1		
			
O3—S1—C1—C12	158.70 (17)	C12—O1—C11—C2	0.2 (2)
O2—S1—C1—C12	28.3 (2)	C12—O1—C11—C10	−179.3 (2)
C14—S1—C1—C12	−85.93 (18)	C3—C2—C11—O1	179.30 (17)
O3—S1—C1—C2	−25.7 (2)	C1—C2—C11—O1	0.2 (2)
O2—S1—C1—C2	−156.10 (19)	C3—C2—C11—C10	−1.1 (3)
C14—S1—C1—C2	89.7 (2)	C1—C2—C11—C10	179.8 (2)
C12—C1—C2—C11	−0.6 (2)	C9—C10—C11—O1	179.38 (19)
S1—C1—C2—C11	−176.64 (17)	C9—C10—C11—C2	−0.1 (3)
C12—C1—C2—C3	−179.4 (2)	C11—O1—C12—C1	−0.7 (2)
S1—C1—C2—C3	4.6 (4)	C11—O1—C12—C13	177.91 (19)
C11—C2—C3—C4	−178.3 (2)	C2—C1—C12—O1	0.8 (2)
C1—C2—C3—C4	0.4 (4)	S1—C1—C12—O1	177.41 (14)
C11—C2—C3—C8	1.3 (3)	C2—C1—C12—C13	−177.3 (3)
C1—C2—C3—C8	180.0 (2)	S1—C1—C12—C13	−0.7 (4)
C2—C3—C4—C5	179.7 (2)	O3—S1—C14—C19	38.53 (17)
C8—C3—C4—C5	0.1 (3)	O2—S1—C14—C19	167.16 (15)
C3—C4—C5—C6	0.3 (3)	C1—S1—C14—C19	−78.17 (16)
C4—C5—C6—C7	−0.1 (3)	O3—S1—C14—C15	−141.32 (15)
C4—C5—C6—Br1	179.40 (17)	O2—S1—C14—C15	−12.69 (18)
C5—C6—C7—C8	−0.6 (3)	C1—S1—C14—C15	101.98 (16)
Br1—C6—C7—C8	179.98 (16)	C19—C14—C15—C16	0.7 (3)
C6—C7—C8—C9	−179.0 (2)	S1—C14—C15—C16	−179.40 (16)
C6—C7—C8—C3	0.9 (3)	C14—C15—C16—C17	0.5 (3)
C4—C3—C8—C7	−0.7 (3)	C15—C16—C17—F1	179.2 (2)
C2—C3—C8—C7	179.70 (18)	C15—C16—C17—C18	−1.5 (4)
C4—C3—C8—C9	179.25 (19)	F1—C17—C18—C19	−179.53 (19)
C2—C3—C8—C9	−0.4 (3)	C16—C17—C18—C19	1.1 (3)
C7—C8—C9—C10	179.0 (2)	C17—C18—C19—C14	0.2 (3)
C3—C8—C9—C10	−0.9 (3)	C15—C14—C19—C18	−1.1 (3)
C8—C9—C10—C11	1.2 (3)	S1—C14—C19—C18	179.04 (15)

### Hydrogen-bond geometry (Å, °)

**Table:**

Cg1 is the centroid of the C14–C19 4-fluorophenyl ring.

**Table:**

*D*—H···*A*	*D*—H	H···*A*	*D*···*A*	*D*—H···*A*
C5—H5···O2^i^	0.95	2.57	3.312 (3)	135.
C18—H18···O2^ii^	0.95	2.36	3.266 (3)	160.
C19—H19···O3^i^	0.95	2.57	3.113 (2)	117.
C10—H10···Cg1^iii^	0.95	2.69	3.609 (2)	152.

Symmetry codes: (i) −*x*+1/2, *y*+1/2, −*z*+1/2; (ii) *x*, *y*+1, *z*; (iii) *x*+1/2, −*y*+1/2, *z*+1/2.
